# A Relational View of Psychological Empowerment and Sense of Community in Academic Contexts: A Preliminary Study

**DOI:** 10.3390/bs9060065

**Published:** 2019-06-18

**Authors:** Ignacio Ramos-Vidal

**Affiliations:** 1 Departamento de Psicología Social, Facultad de Psicología, Universidad de Sevilla, 41018 Sevilla, Spain; iramos5@us.es; 2 Research Group CAVIDA, Escuela de Ciencias Sociales y Humanas, Pontifical Bolivarian University, Montería, Córdoba, Colombia

**Keywords:** classroom, ego-centric networks, empowerment, sense of community, social network analysis, socio-centric networks, university students

## Abstract

Scholars need to pay attention to understand the factors that shape the interactions between individuals and social groups. Constructs like Psychological Sense of Community (PSoC) and Psychological Empowerment (PE) are powerful constructs used to evaluate the antecedents and the consequences of individual attachment to social settings. In parallel, recent advances in network analysis show that the position occupied within whole networks and ego-centric networks are relational factors that affect the subjective perception of membership to social groups. Studies that are conducted in organizational and community settings show strong associations between PSoC and PE. However, these connections have rarely been evaluated within natural settings such as the classroom context. On the other hand, although the theoretic basis of PSoC and PE claims that both processes are formed in a relational way, there are few studies that empirically evaluate the effects of social connectedness on the emergence of PSoC—referred to the classroom—and PE referred to academic-task development. The aim of this research is to determine the effects that the position occupied in formal and informal exchange networks induce on PSoC and PE dimensions. Sixty-four students enrolled in a master degree program (women = 68.8%, Mean age = 26.09, SD = 3.88) participated in this cross-sectional study. Multivariate analyses and network analyses were performed to test the hypotheses under study. The main research finding is that PSoC and PE are synergistic constructs that mutually shape to each other. In relational terms, by sending several nominations in informal networks, it is possible to generate notable impacts on some PSoC dimensions, while receipt of a wide number of nominations in formal contact networks is associated with high levels of PE. In addition, individuals who present high levels of PE are located in the core of formal exchange networks. These results are discussed in order to design actions to increase PSoC and PE in postgraduate academic settings.

## 1. Introduction

Most disciplines within social sciences (i.e., Social Psychology, Anthropology, Sociology, etc.) are interested in understanding the linkages that connect individuals and groups with the social contexts in which they inhabit. Sub-disciplines as the Community Psychology propose strong theories and constructs that focus on evaluating the different forms that those social ties may exhibit [1]. Some theories are focused on explaining the effects of social connectedness on well-being, quality of life, health-related outcomes, and individual performance in groups, organizations, and communities [2,3,4,5].

One of the theoretical models more extensively applied to analyze the connections of individuals to social groups is the Psychological Sense of Community (PSoC) [6]. This construct is widely applied because it offers a strong and comprehensive basis to identify those factors related to the perceptions that individuals develop regarding member feelings in social groups. Although the definitions of community vary notably depending on the study context and the research question to response, there is a basic consensus that considers community in a dual and usually overlapped sense: (a) community understood as a geographic demarcation (i.e., neighborhoods), and (b) community understood as the relationships that bind individuals and groups without having to take into account the physical space in which the interactions occurs (e.g., online communities) [7]. In both cases, the substantive element that shapes the degree of integration of individuals in a community or social group is defined by the ties they maintain with other members of that community or social group. This fact highlights the inherent interactional nature of social belonging to both local and relational communities.

The importance of PSoC lies on the effects that this construct is promoted by catalyzing other processes like the involvement in community activities and the activation of altruistic and prosocial behaviors [8,9]. Recent studies were centered on evaluating a sense of belonging in academic contexts. This puts in the value as the role played by the PSoC activating positive effects at individual and collective level. Freeman, Anderman and Jensen [10] evaluated PSoC at the class-level and campus-level in a university context. Their results show strong associations between students’ sense of class belonging and the level of intrinsic motivation, self-efficacy, and the centrality conferred to academic task development. A study developed in a virtual academic community discovered the positive impacts that PSoC produce on shared-knowledge behaviors [11]. On the other side, some studies identify the effects of the social climate on PSoC, which shows the importance of considering the intrinsic relational component when sense of belonging is evaluated [12,13].

The theoretical connections between social networks and PSoC have been shown in some proposals [14]. However, to date, few studies have developed empirical analysis to evaluate the associations between social relationships and PSoC in academic contexts. Dawson [15] examines this topic showing that individuals occupying central positions in classroom social networks experience the highest levels of PSoC, while members located in bridging positions (that is, acting as intermediaries between groups) present lower levels of PSoC. This result suggests that an individual perception of social inclusion in groups is contingent on the relationships established with group members.

Social Network Analysis (SNA) offer a plethora of methods, instruments, and measures to evaluate both: (a) the whole structure of social contexts, which are reflecting a collective-level phenomena (i.e., relationships of students in a classroom), and (b) the position occupied by actors in the networks evaluated, which shows individual-level phenomena (i.e., identifying isolated or well-connected students). At the collective level, groups characterized by showing a large number of connections between the members are considered cohesive structures, and the members of those groups tend to exhibit high PSoC, which may produce positive impacts at the group level. For example, the work of van den Bos, Crone, Meuwese, and Güroğlu [16] point out the positive consequences of group cohesiveness at the class level. Their study shows that density (the proportion of real versus possible contacts in a social network), is associated with the emergence of prosocial behaviors and trust arising between classmates. At the same time, relational cohesiveness inhibits aggressions and the involvement in anti-social activities among classmates. Systematic analysis of relational factors, which determine the emergence of PSoC and PE in academic contexts, is needed in order to design and implement actions (within classroom and at an institutional level) that aim to increase the attachment of individuals to academic settings and the perception of autonomy and control over academic task development. By activating PSoC and PE, it is possible to (a) increase social connectedness, (b) activate pro-social behaviors, (c) reduce dropout intention, and (d) promote collaborative dynamics [8,9,10,11,12,15,16].

The positive impacts of social connectedness at an individual level and at collective level are explained by several psychosocial mechanisms. On the one hand, social connectedness reflects trust, reciprocity, and the presence of some common goals by group members. These processes facilitate the interplay among actors [17,18,19]. On the other hand, there are antecedents related to the attributes of group members, since the perception of similarity among group members (e.g., homophily) triggers (a) social interactions, (b) selection processes, and (c) influence mechanisms that shape the social network structure [20,21,22].

At an individual level, the analysis is focused on two main areas: (a) to evaluate the local net of direct connections that surround an individual within a social context, referring, in this case, to the egocentric network analysis [23], and (b) to analyze the nominations received or sent by a network member, being common, in this case, to evaluate centrality measures like indegree, outdegree, and betweenness centrality [24]. At a collective level, the analysis is centered on evaluating whole network parameters like density, homophily, reciprocity, centralization, and the number of clusters in which the network could be fragmented [25]. This kind of analysis is useful to compare various whole networks such as different classrooms in the same school. However, it is also applied to compare different networks composed by the same actors such as when the same classroom is analyzed, but evaluates different social interactions like information exchange and a social support provision [26]. SNA literature refers to egocentric networks when the focus is centered on evaluating the relationships maintained by a subject (ego) with a subset of actors (alters), which are members of the whole network structure under study (i.e., classroom). In this case, the objective is to evaluate the impact that Ego is experienced by being embedded in a local sub-network. When the analysis is focused on the total number of connections submitted and receipt by an actor within whole networks, the right way to know the position occupied by an individual is through centrality measures. With respect to the content of relationships within academic contexts, scholars suggest that it is advisable to differentiate among formal relationships (those that are based on interactions to develop academic tasks, to collaborate in group activities or for preparing exams) from informal relationships (which denotes interaction patterns without academic settings such as by going shopping or developing sport activities).

Nevertheless, PSoC is not the only process that serves to understand the connections between individuals and their social environment. Psychological Empowerment (PE) is a key construct to develop an accurate knowledge about how the connections are maintained by individuals in a social context and how they lead to positive consequences for individuals and groups. In a broad sense, PE refers to the acquisition of control and mastery in a particular aspect of life, which is important for a person [27]. The increase of self-efficacy, perceived control, and autonomy constitutes some of the essential signs when PE arise [28,29]. There are a wide variety of research areas that have applied the PE framework including community intervention, health education, programs design, and work and organizational research, among others [30,31,32,33,34].

One of the key features of PE is the intense conceptual and applied connections this construct maintains with PSoC. Several studies state the importance to simultaneously evaluate PE and PSoC, considering that both processes jointly evolve [35]. Another communality between PE and PSoC is that both are characterized by an interactional configuration. The relational view of PE and PSoC is supported by the idea that social relationships constitute a fundamental mechanism to increase the perceived control over social environments (PE), and for feeling member of groups and communities (PSoC). Some theoretical studies stress that the only way to empower people is by promoting relationships that provide access to social resources [36].

In order to disentangle the associations between PE, PSoC, and the relational context, and to know how relationships between classmates determine the level of both constructs in academic contexts, this study is designed to test the next hypotheses.

**Hypothesis 1** **(H1):** *The relationship between the level of PE related to academic task development, and the PSoC at class-level experienced by university students of a master degree program, will be characterized by strong and synergistic associations.*

**Hypothesis 2** **(H2):** *The level of PE related to academic task development and the PSoC at class-level, will be both determined by indegree and outdegree of individuals within formal and informal exchange networks in the classroom. This hypothesis is subdivided in four different sub-hypotheses depending on the kind of relationship (formal and informal) evaluated and the level of analysis (whole and egocentric).*

**Hypothesis 2a** **(H2a):** *Indegree and outdegree centrality of individuals in the whole network of formal contacts will determine the level of PE related to academic task development.*

**Hypothesis 2b** **(H2b):** *Indegree and outdegree centrality in the whole network of informal contacts will determine the level of PSoC at the class-level.*

**Hypothesis 2c** **(H2c):** *Indegree and outdegree centrality in the egocentric network of formal contacts will determine the level of PE related to academic task development.*

**Hypothesis 2d** **(H2d):** *Indegree and outdegree centrality in the egocentric network of informal contacts will determine the level of PSoC at the class-level.*

**Hypothesis 3** **(H3):** *The level of PE related to academic task development and the PSoC at the class-level will be conditioned so that students occupy core or peripheral positions in formal and informal whole networks. Considering the role of formal and informal relationships as triggers of PE and PSoC, the next four sub-hypotheses are proposed.*

**Hypothesis 3a** **(H3a):** *Individuals presenting high levels of PE related to academic task development will be positioned in the core of the whole network of formal contacts.*

**Hypothesis 3b** **(H3b):** *Individuals presenting high levels of PE related to academic task development will be positioned in the core of the whole network of informal contacts.*

**Hypothesis 3c** **(H3c):** *Individuals presenting high levels of PSoC at class-level will be positioned in the core of the whole network of formal contacts.*

**Hypothesis 3d** **(H3d):** *Individuals presenting high levels of PSoC at class-level will be positioned in the core of the whole network of informal contacts.*

## 2. Materials and Methods

### 2.1. Participants

Sixty-four students enrolled in a Master Degree program in Human Resources Management of a public university in Spain participated in this study. The majority of participants are women (n = 44, 68.8%) and the average mean age of the participants is 26.09 years (SD = 3.88). The sample is highly diverse according to the participants’ country of origin (37.5% are foreigners). In the first academic course evaluated, the master degree classroom included 33 students, and, in the second course, the size of the classroom evaluated was 31 students. Due to the preliminary nature of this research, we opt to access an intentional sample of convenience.

### 2.2. Procedure

During two consecutive academic courses, participants fulfilled different instruments to evaluate the constructs under study. All participants signed an informed consent and the research team committed to preserve the anonymity of the respondents and the aggregate use of the data. The study was conducted in accordance with the Declaration of Helsinki. The identification code of the project is: 203-01/17/G-003 (2018/2019).

### 2.3. Instruments

To evaluate the PSoC at the class-level, the second version of the Sense of Community Index (SCI-II) developed by Chavis, Lee, and Acosta was applied [37]. The SCI-II includes 24 items rated on a Likert scale from 1 to 4, where 1 is not in agreement and 4 is in complete agreement (Example of item: *“Being a member of this class is part of my identity”*). The SCI-II was designed to evaluate the multidimensional model proposed by McMillan and Chavis [6]. The dimensions that shaped the PSoC are Reinforcement of Needs, Membership, Influence, and Shared Emotional Connection. The whole scale has optimal psychometric properties (α = 0.84). The average score of the complete scale was 2.76 (SD = 0.38), which would reflect a moderate level of PSoC.

To meet the study objectives, the instrument developed by Spreitzer [38] to measure PE in organizational settings, was adapted to evaluate PE in academic contexts. This scale included 12 items rated on a Likert scale from 1 to 4, where 1 is not in agreement and 4 is in complete agreement (Example of item: *“I have confidence in my ability to do the activities of the master program”*). The model proposed by Spreitzer [38] was characterized by four dimensions that evaluate the degree of domain on different task-related behaviors. The dimensions are Meaning, Competence, Self-determination, and Impact. The instrument exhibited acceptable psychometric properties (α = 0.77). The average score of the complete scale was 2.96 (SD = 0.39), which reflected a moderate level of PE.

To analyze the formal and informal social networks in the classroom, a sociocentric instrument based on previous studies was designed [26,39]. Appendix B describes the instrument designed to evaluate socio-centric networks referred to formal and informal contacts.

Regarding the formal contact network, participants have to nominate the classmates with whom they have collaborated to develop academic activities related to the master program. In the case of the informal contacts network, participants have to nominate classmates with whom they have interacted outside the academic context to develop non-academic activities such go shopping, practice sports, go to the cinema, etc.

### 2.4. Description of Network Variables

#### 2.4.1. Structural Measures

Cohesion parameters were analyzed to evaluate the whole network structure [40]. *Density* showed the total number of ties divided by the possible number of social ties in a network. *Homophily* described the tendency of actors to maintain contacts with other actors with whom they perceived similar features, which is referred to the educational level [41]. *Transitivity* offered information regarding the percentage of fully-connected triads in a given network. *Reciprocity* was expressed as a percentage and reflected the proportion of mutual nominations between each dyad. *Subgroups* analysis showed the number of clusters in which a social network was structured [42].

#### 2.4.2. Centrality or Positional Measures

Centrality measures were analyzed to evaluate the position occupied by each actor in formal and informal networks. For non-symmetric data, the indegree of a vertex *u* is the number of ties received by *u* and the outdegree is the number of ties initiated by *u* [24]. Indegree centrality refers to the mentions receipt by actors in a particular kind of social relationship. Outdegree centrality shows the number of nominations sent by an actor to other members of the classroom. Indegree centrality is considered an indicator of individual prestige and prominence within social groups, while outdegree identify actors that are powerful due to the amount of direct links they maintain with other network members (i.e., ability to influence on group members attitudes and behaviors through direct ties).

**Figure 1:**
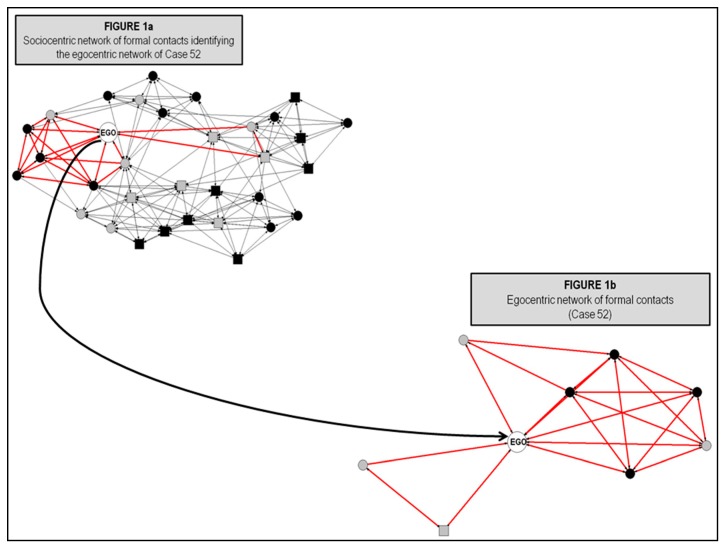
Isolation of an egocentric network from a sociocentric network of formal contacts.

To analyze the centrality of individuals in both whole networks (formal and informal), an egocentric analysis of the individual centrality within their local sub-networks was performed. The direct and indirect ties that surround each actor (Ego) were isolated from the whole networks with the purpose to get the individuals’ egocentric network. Once the egocentric network of each actor was isolated, then the centrality measures were calculated such as in the usual way performed in sociocentric networks (see Marsden [43] for a theoretical and technical explanation). Figure 1 offers a visual explanation of the procedure performed.

This sort of multilevel analysis is useful to differentiate among the centrality within the entire network (whole classroom) and the local relational environment of subjects (egocentric network). As some authors suggest, the combination of egocentric and sociocentric analysis is especially pertinent for linking the micro level and the macro level processes that shape the embeddedness of individuals within social groups [43,44].

### 2.5. Data Analysis

Four adjacent socio-matrices were created using UCINET (v. 6.66) [45]. The outputs analyzed are two socio-matrices per kind of relationship (formal and informal), and per academic course (first and second year). Reciprocity, density, homophily (according to sex), and number of clusters were calculated to characterize the whole network structure. In a second step, indegree centrality and outdegree centrality were calculated for each network to determine the individual centrality of the participants in formal and informal contact networks. The categorical partition model proposed by Borgatti and Everett [46] was executed to identify the actors occupying central and peripheral positions in formal and informal networks. This model served to fragment the network into two subsets named core and periphery. As Borgatti and Everett states [46] (p. 377–378), “*core nodes are adjacent to other core nodes, core nodes are adjacent to some periphery nodes, and periphery nodes do not connect with other periphery nodes.*” In simple terms, this analysis allowed the differentiation between one area of well-connected actors (core) and one area of poor-connected actors (periphery).

**Figure 2:**
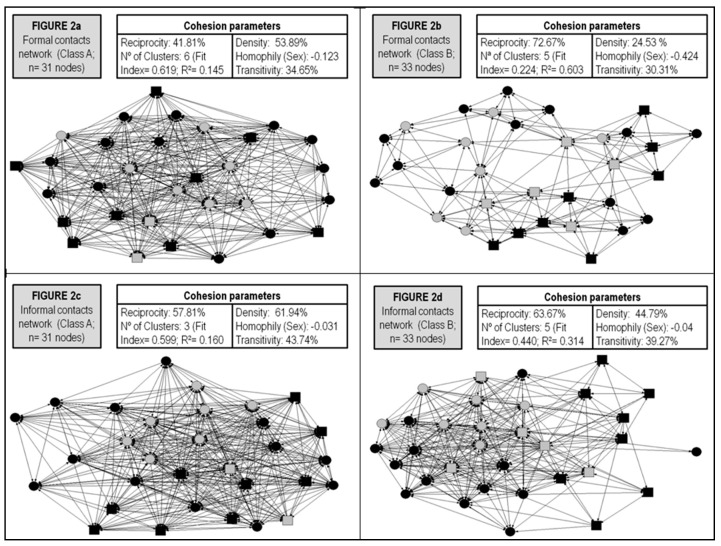
Structural properties of the four networks under study. *Note 1:* The color of nodes represents the sex of participants (Black nodes = Women. Grey nodes = Men). *Note 2:* The shape of nodes represent the participants’ origin (Circle = Locals. Square = Foreigners).

Different analytic procedures, including visual representation of the four networks evaluated, were developed to test the hypotheses under study. To test the first hypothesis, the bivariate correlation analysis was performed to observe the associations among PE dimensions and PSoC dimensions. To evaluate the second hypothesis and sub-hypotheses, which have focused on analyzing the effects of centrality measures on PE and PSoC, several linear regression models were performed. To test H3, a set of non-parametric Kruskal-Wallis tests were carried out [47]. Statistical analyses were performed using the SPSS ® package (v.25) (IBM Corp., Armonk, NY, USA). Figure 2 illustrates the structural features of the four classroom networks evaluated.

## 3. Results

### 3.1. Testing Hypotheses

#### 3.1.1. H1

The first hypothesis was designed to ascertain if there are associations among the dimensions that shape PE and PSoC in the academic context under study. To analyze the relationships between the dimensions of both constructs, bivariate correlation analysis was performed. The results are shown in Table 1.

Our results highlight the interplay between both constructs. Intense correlations (r = 0.456, *p* < 0.0001) among the mean score of both variables were observed. However, not all dimensions present positive or intense correlations. Impact and Meaning (PE) presents intense connections with all PSoC dimensions. In contrast, Competence and Self-determination shows no correlation with PSoC dimensions. This evidence points out the synergy between PE and PSoC in the academic context evaluated, which offers partial support to the first hypothesis.

#### 3.1.2. H2

To test the second hypothesis and sub-hypotheses, several multiple linear regression models were performed (See Appendix A for a description of the variables included in the models). In short, the dependent variables are the average score of PE and its sub-dimensions (Meaning, Competence, Self-determination, and Impact), and the average score of PSoC and it sub-dimensions (Reinforcement of needs, membership, influence, and shared emotional connection). The independents are indegree and outdegree centrality in the whole network of formal and informal contacts, and the same variables in the egocentric –isolated- network of formal and informal contacts. Table 2 shows the regression coefficients.

The regression analyses show a partial support for the second hypothesis and sub-hypotheses. H2a have no support due to none of the proposed regression models fitting at the minimum level of statistical significance (*p* < 0.05). In consequence, H2a should be rejected.

In contrast, H2b is partially supported by the data analysis. The dimension membership (PSoC) is largely predicted by outdegree centrality (number of nominations submitted) in the whole network of informal contacts (β = 0.524, *p* < 0.001), whereas indegree centrality (received nominations) made no contributions to explain the dependent. There are two additional models in which outdegree exert a notable incidence on the variability of reinforcement of needs and the complete PSoC scale, even though the regression coefficients of those models do not completely adjust. This result shows the important role of maintaining an active role in informal relationships contexts, due to the catalytic effect that those informal contacts promote on important dimensions of PSoC, such as membership and reinforcement of needs.

In attention to H2c, the results suggest that the level of connectedness, in terms of the nominations received and sent, within the egocentric formal network, induces a modest effect on some PE and PSoC dimensions. Even though only one regression model shows a good fit (model 23), this result suggests that being nominated by other members of the Egos’ focal network seems to be an important factor to activate the perception of competence for academic task performance. Receiving nominations in the formal contacts network shows that Ego is perceived by the members of their local sub-network as an information resource for developing academic activities. However, there is some support for the H2c considering that only one of the 10 regression models proposed to test these sub-hypotheses exhibits an acceptable adjustment.

Some interesting results are identified regarding to H2d. In this case, PSoC dimensions present notable variations exerted by the effects of centrality measures within the egocentric informal network. Nevertheless, PE dimensions seem not to be impacted in any way by centrality parameters in the egocentric informal network. Table 2 shows how key PSoC dimensions (reinforcement of needs and membership) are widely influenced by outdegree centrality of actors within informal local networks. Conversely, indegree centrality shows no effects on dependent variables. This result shows that active individuals reporting several informal contacts with other members of their local network, are characterized by high levels of sense of belonging to their classroom, and perceive that their social demands are satisfied. The nominations to other ego-network members are likely acting as the main trigger of PSoC. Therefore, the H2d is partially supported by models 36–38 (see Table 2).

#### 3.1.3. H3

The third hypothesis was proposed to test to what extent the level of PE and PSoC explain the position occupied by individuals within the network structure. The results presented in Table 3 offer support to sub hypothesis H3a. Individuals located at the core of the formal exchange network exhibit high PE levels (complete scale) compared with subjects located at the periphery of the same network (χ^2^ = 4.115, *p* < 0.038). An identical trend is identified in the dimension impact of PE (χ^2^ = 7.617; *p* < 0.004). In contrast, there are no associations between the positions occupied in the informal exchange network and the level of PE. As a consequence, H3b should be rejected.

The next sub hypotheses were designed to know if the level of PSoC referred to the classroom explains the position held by actors within formal (H3c) and informal (H3d) exchange networks. The analysis executed shows that the level of PSoC does not explain the location of individuals at the core/periphery of the two kinds of networks evaluated. Thus, we must discard H3c and H3d. In the next section, the main research findings and the limitations of this investigation are discussed.

## 4. Discussion

The purpose of this paper is two-fold. On the one hand, the purpose is to show the conceptual and empirical connections between PE and PSoC in academic contexts. On the other hand, the purpose was to determine if the relationships maintained by students within the formal and informal exchange networks in the classroom, determine the level of PE regarding academic tasks and the PSoC is referred to the classroom.

The results show that PE and PSoC are deeply interrelated and are mutually strengthened. This evidence is constant with previous research posits that PE and PSoC are different but interrelated processes [35,48,49,50]. Nevertheless, the associations between PE and PSoC have been commonly tested in community settings, which do not commonly demonstrate their associations in natural interaction contexts as classrooms in postgraduate training programs. For example, in investigations conducted in organizational settings, it is common that competence and self-determination presents remarkable associations with PSoC dimensions. However, in the academic context analyzed, both dimensions are independent regarding PSoC dimensions.

However, formal and informal relationships do not have the same impact on all the dimensions that shape PSoC and PE. There are remarkable associations between the individual centrality of participants in the network of informal contacts and core dimensions of PSoC. The core dimension of PSoC is membership. Several studies within the field of Community Psychology and Psychology of Groups shows that the subjective perception of being part of social groups—membership—is an essential factor for explaining the degree of attachment to social structures [6,7,8,13,14,51]. In this sense, our results show that, by nominating other classmates in the informal contacts network, it may produce a triggering effect activating the feeling of membership to a classroom (r = 0.46, *p* < 0.001), and also produce a small impact on the complete construct (r = 0.36, *p* < 0.05). The same tendency is observed when the closer structure of contacts (called ego-centric network) is analyzed, which surrounds individuals in natural social settings [23,52]. Nonetheless, this association is not observed when the connections between the individual centrality in the formal contact network and the PSoC dimensions are explored. This result suggests that PSoC is mainly affected when individuals maintain an active strategy of informal contacts by nominating several contacts within the classroom. Inversely, to activate PSoC, the nominations received in informal and formal contacts network are not important in the context evaluated. This shows that, in the academic context analyzed, the reciprocity deploys a residual role to explain the emergence of PSoC, which shows that informal relationships constitute a key variable to explain the subjective attachment to social groups.

A differential effect is identified if we focus on the effects produced by social connectedness on PE. In contrast to PSoC, the activation of PE requires the occurrence of a formal relationship. Nevertheless, the key factor here is not to submit or to nominate several contacts, but to receive nominations in the network of formal contacts. The fact of receiving nominations in networks that denotes academic or task-related activities constitutes a signal of mastery, and, perhaps, this relational activity make it feasible to precipitate or activate some PE dimensions such as perceived competence. Another aspect to mention is that this association is more intense in egocentric networks when compared with the centrality in the whole networks of formal contacts. This could be explained because the empowerment process implies the acquisition of control over the local environment and, in relational terms, the first step to control the local context is experiencing control on the own egocentric network. In this study, the number of nominations received in the egocentric formal contact networks, impact the perceived competence in academic task development (r = 0.35, *p* < 0.001). Other interesting results is that individuals who occupy core positions within the complete network of formal contacts, reports the highest levels of PE (χ^2^ = 4.115, *p* < 0.038), and perceive that their actions in this context produce notable impacts on the whole classroom (χ^2^ = 7.617, *p* < 0.004). This evidence suggests that well-connected actors such as those maintaining an active strategy in formal relationships with other classmates, experience high levels of PE compared with less active individuals in relational terms.

Future lines of research have to devote attention to some of the topics mentioned below. The studies may be focused on exploring the interplay between formal and informal relational contexts. This implies the need to empirically evaluate how certain kind of relationships (such as the mere recognition of a person in a classroom), could be a precursor of other types of relationships (such as informal contacts to develop academic tasks). In this sense, there are well-established methods in SNA, as the family of Quadratic Assignment Procedure (QAP) tools, that could be a great ally to analyze the degree of overlapping between different networks composed by the same actors [53,54]. At the same time, future research needs to evaluate the role exerted by other stakeholders that could be affecting the relationships maintained by the students in the classroom in a variety of ways. For example, teachers and academic staff may facilitate or inhibit the interactions between students within the classroom (such as promoting interactions through group tasks), and it is likely that the constriction or the promotion of interactions will affect the network structure and the PSoC and PE processes. The assessment of personality traits could offer valuable clues to understand the relational tactics developed by students to increase control over their social environment [55,56,57]. In recent years, a wide body of literature has growth with the objective to determine the influence that some personality variables (e.g., extroversion and neuroticism) exert on the variability of the network structure and on individual performance in social networks. Lastly, the comparative analysis of behavioral—real—social networks versus the cognitive—perceptual—evaluation of social networks could be a promising field of research for understanding the relational factors impacting the degree of power that individuals achieve in a variety of social contexts [58,59,60].

Some limitations should be discussed in order to clarify the contributions of this work to the existing literature. First, the small—and intentional—sample makes it difficult to extrapolate the results to other academic settings without a previous contextual adaptation. Second, the study design is cross-sectional. As a consequence, our results can only support covariance associations among the variables under study but no causality relationships. Third, to evaluate PSoC and PE in the academic context analyzed, we adapted instruments developed to evaluate both constructs in community and organizational settings, respectively. Due to this fact, specific instruments need to be designed and validated for evaluating PSoC and PE in academic contexts.

## Figures and Tables

**Table 1:** Matrix of bivariate correlations among the PE and PSoC dimensions and centrality measures in formal and informal networks at whole and egocentric levels.

Variables	M	SD	Min.	Max	Kurtosis	1	2	3	4	5	6	7	8	9	10	11	12	13	14	15	16	17	18
**1.** PE ^†^	2.96	0.39	2	3.75	−0.161																		
**2.** Meaning (PE)	3.36	0.56	2	4	−0.155	0.59 **																	
**3.** Competence (PE)	3.16	0.61	1.66	4	−0.375	0.73 **	0.25 *																
**4.** Self-determination (PE)	2.85	0.58	1.66	4	−0.535	0.58 **	0.01	0.31 *															
**5.** Impact (PE)	2.49	0.62	1.33	4	−0.547	0.74 **	0.35 **	0.35 **	0.23														
**6.** PSoC ^†^	2.76	0.38	2.12	3.66	−0.621	0.45 **	0.35 **	0.18	0.10	0.56 **													
**7.***Reinforc.* (PSoC)	2.79	0.42	2	3.83	−0.459	0.39 **	0.30 *	0.11	0.01	0.45 **	0.73 **												
**8.** Membership (PSoC)	2.52	0.43	1.66	3.33	−0.732	0.41 **	0.16	0.20	0.25	0.44 **	0.70 **	0.42 **											
**9.** Influence (PSoC)	2.61	0.45	1.83	3.5	−0.873	0.36 **	0.22	0.18	0.03	0.49 **	0.79 **	0.40 **	0.42 **										
**10.***Shared Em.* (PSoC)	3.11	0.61	1.83	4.5	−0.368	0.35 **	0.38 **	0.11	0.03	0.42 **	0.87 **	0.53 **	0.43 **	0.66 **									
**11.** Outdegree (Formal Cont. *Whole Net.*)	0.31	0.15	0.13	1.03	7.08	0.06	0.05	−0.16	0.09	0.17	0.15	0.11	0.04	0.12	0.18								
**12.** Indegree (Formal Cont. *Whole Net.*)	0.31	0.12	0.13	0.81	3.01	0.08	−0.06	0.14	0.03	0.08	0.05	−0.04	−0.01	0.17	0.07	0.34 **							
**13.** Outdegree (Inform. Cont. *Whole Net.*)	0.39	0.21	0	1.03	0.63	0.17	0.04	0.08	0.06	0.25	0.31 *	0.21	0.46 **	0.09	0.23	−0.06	−0.01						
**14.** Indegree (Inform. Cont. *Whole Net.*)	0.38	0.17	0.06	0.78	−0.98	0.06	−0.09	0.17	0.04	0.05	0.15	0.11	0.17	0.15	0.10	−0.11	0.11	0.62 **					
**15.** Outdegree (Formal Cont. *Ego-Net.*)	0.78	0.17	0.36	1	−0.42	0.03	0.07	−0.07	0.03	0.06	0.16	0.18	0.19	0.01	0.13	0.17	−0.52 **	0.21	0.09				
**16.** Indegree (Formal Cont. *Ego-Net.*)	0.81	0.16	0.2	1	1.57	0.05	−0.07	0.35 **	−0.05	−0.09	−0.18	−0.17	−0.17	−0.06	−0.15	−0.63 **	0.01	0.13	0.33 **	−0.21			
**17.** Outdegree (Inform. Cont. *Ego-Net.*)	0.73	0.22	0	1	1.74	0.07	0.04	0.03	0.01	0.11	0.23	0.26 *	0.40 **	−0.03	0.13	−0.25 *	−0.20	0.68 **	0.34	0.29 *	0.18		
**18.** Indegree (Inform. Cont. *Ego-Net.*)	0.79	0.17	0.15	1	2.2	−0.03	−0.06	0.12	−0.04	−0.10	0.02	0.11	−17	0.10	0.04	−0.07	0.03	−0.33 **	0.40 **	−0.01	0.23	−0.43 **	

Note: * = *p* < 0.05; ** = *p* < 0.001; ^†^ Complete scale mean; *Reinforc.* = Reinforcement of needs; *Shared Em.* = Shared emotional connection; *Whole Net.* = Whole network; *Ego-Net.* = Egocentric network.

**Table 2:** Coefficients of regression models.

Model		Regression Coefficients										
		*β (Outdeg)*	*t (Outdeg)*	*p (Outdeg)*	*β* *(Indeg)*	*t* *(Indeg)*	*p* *(Indeg)*	*R* ^2^	*F*	*B*	*p*	*Dependent Variable*
**H2a**	1	0.035	0.245	0.808	0.071	0.497	0.621	0.008	0.229	2.650	0.796	PE (Complete scale mean)
	2	0.091	0.641	0.524	−0.098	−0.696	0.489	0.011	0.328	3.400	0.721	Meaning (PE)
	3	−0.247	−1.808	0.076	0.239	1.747	0.086	0.075	2.314	3.091	0.108	Competence (PE)
	4	0.098	0.690	0.443	0.000	0.002	0.999	0.010	0.275	2.738	0.760	Self-determination (PE)
	5	0.160	1.144	0.258	0.031	0.220	0.827	0.030	0.889	2.247	0.417	Impact (PE)
	6	0.149	1.043	0.302	0.002	0.017	0.986	0.022	0.629	2.650	0.537	PSoC (Complete scale mean)
	7	0.147	1.039	0.303	−0.096	−0.680	0.499	0.021	0.593	2.779	0.556	Reinforcement of needs (PSoC)
	8	0.059	0.400	0.691	−0.031	−0.218	0.828	0.003	0.083	2.514	0.920	Membership (PSoC)
	9	0.067	0.477	0.635	0.153	1.098	0.277	0.002	1.048	2.380	0.357	Influence (PSoC)
	10	0.186	1.320	0.192	0.011	0.077	0.939	0.036	1.045	2.873	0.358	Shared emotional con. (PSoC)
**H2b**	11	0.192	1.223	0.226	−0.037	−0.237	0.813	0.030	0.889	2.856	0.417	PE (Complete scale mean)
	12	0.150	0.951	0.345	−0.181	−1.149	0.255	0.025	0.731	3.442	0.486	Meaning (PE)
	13	−0.021	−0.134	0.894	0.184	1.171	0.246	0.030	0.879	2.915	0.421	Competence (PE)
	14	0.053	0.334	0.740	0.014	0.086	0.932	0.004	0.109	2.772	0.897	Self-determination (PE)
	15	0.324	2.116	0.039	−0.127	−0.828	0.411	0.075	2.327	2.294	0.107	Impact (PE)
	16	0.321	2.083	0.042	−0.020	−0.127	0.899	0.096	2.927	2.551	0.062	PSoC (Complete scale mean)
	17	0.212	1.349	0.183	0.001	0.006	0.995	0.045	1.324	2.629	0.274	Reinforcement of needs (PSoC)
	18	0.524	3.670	0.001	−0.113	−0.791	0.432	0.222	7.840	2.218	0.001	Membership (PSoC)
	19	0.011	0.068	0.946	0.148	0.938	0.352	0.024	0.692	2.446	0.505	Influence (PSoC)
	20	0.248	1.587	0.118	−0.029	−0.186	0.853	0.054	1.604	2.867	0.210	Shared emotional con. (PSoC)
**H2c**	21	0.044	0.328	0.744	0.058	0.437	0.664	0.005	0.129	2.775	0.879	PE (Complete scale mean)
	22	0.061	0.453	0.652	−0.065	−0.488	0.627	0.009	0.265	3.385	0.768	Meaning (PE)
	23	−0.018	−0.142	0.887	0.349	2.782	0.007	0.124	4.053	2.165	0.023	Competence (PE)
	24	0.025	0.188	0.851	−0.054	−0.400	0.690	0.004	0.113	2.935	0.893	Self-determination (PE)
	25	0.052	0.387	0.700	−0.088	−0.662	0.510	0.012	0.345	2.617	0.710	Impact (PE)
	26	0.137	1.026	0.310	0.165	−1.237	0.221	0.054	1.559	2.830	0.220	PSoC (Complete scale mean)
	27	0.159	1.205	0.233	−0.147	−1.119	0.268	0.055	1.622	2.798	0.207	Reinforcement of needs (PSoC)
	28	0.167	1.259	0.213	−0.145	−1.091	0.280	0.057	1.677	2.506	0.196	Membership (PSoC)
	29	0.009	0.066	0.948	−0.065	−0.482	0.632	0.004	0.127	2.737	0.881	Influence (PSoC)
	30	0.110	0.827	0.412	−0.139	−1.047	0.300	0.067	1.066	3.223	0.351	Shared emotional con. (PSoC)
**H2d**	31	0.078	0.541	0.591	0.000	0.001	1	0.006	0.176	2.857	0.839	PE (Complete scale mean)
	32	0.028	0.195	0.846	−0.050	−0.346	0.731	0.005	0.129	3.432	0.879	Meaning (PE)
	33	0.108	0.754	0.454	0.171	1.190	0.239	0.026	0.750	2.448	0.477	Competence (PE)
	34	−0.001	−0.009	0.993	−0.049	−0.337	0.737	0.002	0.067	2.981	0.935	Self-determination (PE)
	35	0.068	0.470	0.640	−0.078	−0.541	0.591	0.015	0.437	2.566	0.648	Impact (PE)
	36	0.293	2.054	0.045	0.142	0.995	0.324	0.072	2.121	2.134	0.130	PSoC (Complete scale mean)
	37	0.378	2.765	0.008	0.275	2.012	0.049	0.133	4.279	1.724	0.019	Reinforcement of needs (PSoC)
	38	0.398	2.929	0.005	−0.012	−0.085	0.932	0.162	5.317	1.953	0.008	Membership (PSoC)
	39	0.013	0.089	0.929	0.108	0.748	0.457	0.011	0.309	2.373	0.735	Influence (PSoC)
	40	0.183	1.263	0.212	0.125	0.863	0.392	0.030	0.866	2.374	0.426	Shared emotional con. (PSoC)

Note: Grey background highlights the models with statistical significance.

**Table 3:** Average range of PE and PSoC dimensions differentiating between core and peripheral actors in formal and informal networks.

Variable	Formal Contacts Whole Network							Informal Contacts Whole Network						
	Position	N	Average Range	χ^2^	*p*	CI:99% (Inf.)	CI:99% (Sup.)	Position	N	Average Range	χ^2^	*p*	CI:99% (Inf.)	CI:99% (Sup.)
PE ^†^	Core	28	35.38	4.115	0.038	0.033	0.043	Core	43	31.41	0.412	0.538	0.525	0.550
	Periphery	32	26.23					Periphery	17	28.21				
Meaning (PE)	Core	28	32.59	0.783	0.375	0.362	0.387	Core	43	31.69	0.729	0.393	0.386	0.412
	Periphery	32	28.67					Periphery	17	27.50				
Competence (PE)	Core	28	34.41	2.757	0.099	0.091	0.106	Core	43	31.21	0.262	0.618	0.605	0.631
	Periphery	32	27.08					Periphery	17	28.71				
Self-determination (PE)	Core	28	30.80	0.016	0.902	0.895	0.910	Core	43	29.22	0.839	0.360	0.348	0.372
	Periphery	32	30.23					Periphery	17	33.74				
Impact (PE)	Core	28	37.05	7.617	0.004	0.002	0.005	Core	43	31.91	1.015	0.326	0.314	0.338
	Periphery	32	24.77					Periphery	17	26.94				
PSoC ^†^	Core	26	33.02	2.053	0.162	0.162	0.181	Core	41	31.55	2.065	0.169	0.159	0.178
	Periphery	32	26.64					Periphery	17	24.56				
Reinforcement of needs (PSoC)	Core	27	32.09	0.752	0.400	0.388	0.413	Core	42	32.5	3.144	0.074	0.068	0.081
	Periphery	32	28.23					Periphery	17	23.82				
Membership (PSoC)	Core	26	31.54	0.696	0.412	0.399	0.424	Core	41	31.8	2.641	0.104	0.096	0.112
	Periphery	32	27.84					Periphery	17	23.94				
Influence (PSoC)	Core	28	33.05	1.139	0.284	0.273	0.296	Core	43	30.76	0.033	0.862	0.853	0.871
	Periphery	32	28.27					Periphery	17	29.85				
Shared emotional connection (PSoC)	Core	27	34	2.723	0.092	0.085	0.100	Core	42	31.95	1.900	0.166	0.157	0.176
	Periphery	32	26.63					Periphery	17	25.18				

Note: ^†^ = Complete scale. The grey background highlights models with statistical significance.

## Appendix A

Appendix A describes the dependent and independent variables included in the regression models designed to test the second study hypothesis.

**Table A1:** Description of regression models indicating the hypothesis tested.

Model		Independents	Dependent
**H2a**	1	Outdegree + Indegree (Formal Contact Whole Network)	PE (Complete scale mean)
	2	Outdegree + Indegree (Formal Contact Whole Network)	Meaning (PE)
	3	Outdegree + Indegree (Formal Contact Whole Network)	Competence (PE)
	4	Outdegree + Indegree (Formal Contact Whole Network)	Self-determination (PE)
	5	Outdegree + Indegree (Formal Contact Whole Network)	Impact (PE)
	6	Outdegree + Indegree (Formal Contact Whole Network)	PSoC (Complete scale mean)
	7	Outdegree + Indegree (Formal Contact Whole Network)	Reinforcement of needs (PSoC)
	8	Outdegree + Indegree (Formal Contact Whole Network)	Membership (PSoC)
	9	Outdegree + Indegree (Formal Contact Whole Network)	Influence (PSoC)
	10	Outdegree + Indegree (Formal Contact Whole Network)	Shared emotional con. (PSoC)
**H2b**	11	Outdegree + Indegree (Informal Contact Whole Network)	PE (Complete scale mean)
	12	Outdegree + Indegree (Informal Contact Whole Network)	Meaning (PE)
	13	Outdegree + Indegree (Informal Contact Whole Network)	Competence (PE)
	14	Outdegree + Indegree (Informal Contact Whole Network)	Self-determination (PE)
	15	Outdegree + Indegree (Informal Contact Whole Network)	Impact (PE)
	16	Outdegree + Indegree (Informal Contact Whole Network)	PSoC (Complete scale mean)
	17	Outdegree + Indegree (Informal Contact Whole Network)	Reinforcement of needs (PSoC)
	18	Outdegree + Indegree (Informal Contact Whole Network)	Membership (PSoC)
	19	Outdegree + Indegree (Informal Contact Whole Network)	Influence (PSoC)
	20	Outdegree + Indegree (Informal Contact Whole Network)	Shared emotional con. (PSoC)
**H2c**	21	Outdegree + Indegree (Formal Contact Egocentric Network)	PE (Complete scale mean)
	22	Outdegree + Indegree (Formal Contact Egocentric Network)	Meaning (PE)
	23	Outdegree + Indegree (Formal Contact Egocentric Network)	Competence (PE)
	24	Outdegree + Indegree (Formal Contact Egocentric Network)	Self-determination (PE)
	25	Outdegree + Indegree (Formal Contact Egocentric Network)	Impact (PE)
	26	Outdegree + Indegree (Formal Contact Egocentric Network)	PSoC (Complete scale mean)
	27	Outdegree + Indegree (Formal Contact Egocentric Network)	Reinforcement of needs (PSoC)
	28	Outdegree + Indegree (Formal Contact Egocentric Network)	Membership (PSoC)
	29	Outdegree + Indegree (Formal Contact Egocentric Network)	Influence (PSoC)
	30	Outdegree + Indegree (Formal Contact Egocentric Network)	Shared emotional con. (PSoC)
**H2d**	31	Outdegree + Indegree (Informal Contact Egocentric Network)	PE (Complete scale mean)
	32	Outdegree + Indegree (Informal Contact Egocentric Network)	Meaning (PE)
	33	Outdegree + Indegree (Informal Contact Egocentric Network)	Competence (PE)
	34	Outdegree + Indegree (Informal Contact Egocentric Network)	Self-determination (PE)
	35	Outdegree + Indegree (Informal Contact Egocentric Network)	Impact (PE)
	36	Outdegree + Indegree (Informal Contact Egocentric Network)	PSoC (Complete scale mean)
	37	Outdegree + Indegree (Informal Contact Egocentric Network)	Reinforcement of needs (PSoC)
	38	Outdegree + Indegree (Informal Contact Egocentric Network)	Membership (PSoC)
	39	Outdegree + Indegree (Informal Contact Egocentric Network)	Influence (PSoC)
	40	Outdegree + Indegree (Informal Contact Egocentric Network)	Shared emotional con. (PSoC)

## Appendix B

Description of the instrument to evaluate socio-centric networks in classroom contexts

Name: _________________________________________________________________

Nationality: _____________ Age (years): ______ Gender: M ___ F___ Other_____

**IMPORTANT:** First you must leave empty the row in which your name appears.

Each column represents a different type of relationship (A, B and C):

✓ In column “A” mark with an X the colleagues you recognize by name.
✓ In column “B” mark with an X the colleagues with whom you frequently collaborate in activities related to the master (for example developing group tasks, preparing exams, exchanging academic documents etc.).
✓ In column “C” mark with an X the colleagues with whom you invest your time outside the academic context doing activities not related to the master (for example go shopping, play sports etc.).

**Table A2:** Description of the instrument to evaluate socio-centric networks in classroom contexts.

Nº	Name and Surname	A	B	C
1				
2				
3				
4				
5				
6				
7				
8				
9				
10				
11				
12				
13				
14				
15				
16				
17				
18				
19				
20				
21				
22				
23				
24				
25				
26				
27				
28				
29				
30				

Note: Name and surname information is extracted from the class list.
